# Choline Kinase Alpha and Hexokinase-2 Protein Expression in Hepatocellular Carcinoma: Association with Survival

**DOI:** 10.1371/journal.pone.0046591

**Published:** 2012-10-05

**Authors:** Sandi A. Kwee, Brenda Hernandez, Owen Chan, Linda Wong

**Affiliations:** 1 The Queen's Medical Center, Honolulu, Hawaii, United States of America; 2 University of Hawaii Cancer Center, University of Hawaii at Manoa, Honolulu, Hawaii, United States of America; 3 Department of Surgery, John A. Burns School of Medicine, University of Hawaii at Manoa, Honolulu, Hawaii, United States of America; The University of Hong Kong, China

## Abstract

**Purpose:**

Hexokinase-2 (HK2) and more recently choline kinase alpha (CKA) expression has been correlated with clinical outcomes in several major cancers. This study examines the protein expression of HK2 and CKA in hepatocellular carcinoma (HCC) in association with patient survival and other clinicopathologic parameters.

**Methods:**

Immunohistochemical analysis for HK2 and CKA expression was performed on a tissue microarray of 157 HCC tumor samples. Results were analyzed in relation to clinicopathologic data from Surveillance, Epidemiology, and End-Results Program registries. Mortality rates were assessed by Kaplan-Meier estimates and compared using log-rank tests. Predictors of overall survival were assessed using proportional hazards regression. RESULTS: Immunohistochemical expression of HK2 and CKA was detected in 71 (45%) and 55 (35%) tumor samples, respectively. Differences in tumor HK2 expression were associated with tumor grade (p = 0.008) and cancer stage (p = 0.001), while CKA expression differed significantly only across cancer stage (p = 0.048). Increased mortality was associated with tumor HK2 expression (p = 0.003) as well as CKA expression (p = 0.03) with hazard ratios of 1.86 (95% confidence interval (CI) 1.23–2.83) and 1.59 (95% CI 1.04–2.41), respectively. Similar effects on overall survival were noted in a subset analysis of early stage (I and II) HCC. Tumor HK2 expression, but not CKA expression, remained a significant predictor of survival in multivariable analyses.

**Conclusion:**

HK2 and CKA expression may have biologic and prognostic significance in HCC, with tumor HK2 expression being a potential independent predictor of survival.

## Introduction

Hepatocelllular carcinoma (HCC) is a leading cause of cancer mortality that accounted for an estimated 695,000 deaths world-wide in 2008 [1]. Tumor resection and liver transplantation offer patients with HCC the best chance for long-term survival. However, many patients are disqualified from surgery as a result of already having locally advanced or metastatic HCC. This loss of surgical opportunity emphasizes the value of early detection and accurate staging to improve clinical outcomes in HCC. In this regard, continued advancements in cancer imaging and diagnostics may have a significant bearing on the surgical treatment of this disease.

A substantial amount of data supports hexokinase-2 (HK2) as a molecular target for the diagnosis and treatment cancer [2], [3]. HK2 is a pivotal enzyme in glucose metabolism and catalyzes the rate-limiting step in glycolysis [4]. Hyperglycolysis occurs in many different tumor types and potentially confers a survival advantage to cancer cells [3]. Positron emission tomography (PET) imaging, using fluorine-18 fluorodeoxyglucose (FDG) as a radiopharmaceutical tracer substrate of HK2, capitalizes on this metabolic phenomenon to image and detect cancer [2]. Unfortunately, the results of clinical studies on FDG PET suggest this technique may be less sensitive for detecting HCC than for other cancers [5]–[7].

The overexpression of choline kinase alpha (CKA) in many cancers has also generated interest in phospholipid metabolism as a diagnostic or therapeutic target in oncology [8]–[11]. CKA catalyzes the synthesis of phosphocholine, a phospholipid precursor for cell membrane synthesis that may also play a role in mitogenic signal transduction [8]–[11]. Tumor uptake of radiolabeled choline has proven to correlate with tissue CKA expression in the animal model of viral-induced HCC [12], and the clinical detection of HCC using choline-based PET tracers has been supported in human clinical trials [13]. While CKA holds promise as a molecular target in HCC, there is still limited understanding about its role in liver tumor biology or its association with other clinicopathologic characteristics in HCC.

While not all hepatomas demonstrate hyperglycolysis, tumor glycolytic activity in HCC has been correlated with HK2 expression in tumors and the risk of cancer recurrence [14]–[17]. Less is currently known about the role of choline metabolism in HCC, although there is increasing evidence supporting the prognostic relevance of CKA expression in other cancers [18]–[20]. To investigate HK2 and CKA expression as potential clinicopathologic variables in HCC, we assembled a microscopy array composed of HCC specimens from an institutional tumor tissue repository to allow tumor HK2 and CKA protein expression to be examined in tandem and in relation to clinicopathologic and survival data obtained from National Cancer Institute Surveillance, Epidemiology, and End Results (SEER) program member registries.

## Methods

### Patients and specimens

The University of Hawaii Committee on Human Studies (IRB) approved this study. As this was a retrospective study using archive tissue specimens and State of Hawaii cancer registry data, the IRB waived the need for written informed consent. Formalin-fixed paraffin-embedded (FFPE) tumor specimens from 157 adult cases of HCC were obtained from the Residual Tissue Repository of the University of Hawaii Cancer Center [21], [22]. These samples were derived from cases of HCC diagnosed within our state from the years 1986 to 2009. Only specimens classified under site code C22.0 (liver) and histologic codes 8170–8175 (hepatocellular carcinoma) by the International Clasification of Diseases-Oncology-3^rd^ Edition were selected. These samples were annotated with de-identified clinical, pathologic, and survival data collected by the SEER program member registries within our state. Because of the de-identification process, 5-year age ranges were used for analysis in lieu of actual age. The cancer staging system was based on American Joint Commission on Cancer 7^th^ edition TNM schema [23]. Tumor grade was classified according to Edmondson-Steiner histopathologic grading as grade I (well-differentiated), grade II (moderately differentiated), grade III (poorly differentiated), and grade IV (undifferentiated) [24].

### Tissue Microarray Construction

The methods used for tumor tissue micro-array construction are previously described [22], [25], [26]. Hematoxylin and eosin slides of each tissue specimen block were examined by a surgical pathologist to identify representative areas of tumor tissue. Cylindrical tissue cores measuring 0.6 mm diameter were obtained from the corresponding areas within the tissue blocks and transferred into an array block using a semi-automated tissue-arraying instrument (TMArrayer, Pathology Devices, Westminster, MD). When sufficient tissue was available, up to four replicate tissue cores were taken from each sample and included on the array.

### Analysis of Tissue CKA and HK2 Expression

Immunohistochemical analysis was performed using an automated staining platform (Dako, Inc., Carpinteria, CA, USA). Protein expression of CKA was evaluated using a commercial antibody (choline kinase alpha rabbit polyclonal antibody, Sigma) in accordance with the manufacturer's protocol and an antibody dilution to 1∶140. HK2 was evaluated using a commercial antibody (hexokinase II rabbit monoclonal antibody, Cell Signaling Technology, Inc., Danvers, MA, USA). The manufacturer's protocol was followed with antibody dilution adjusted to 1∶100. Detection was conducted using a biotinylated secondary anti-rabbit IgG and avidin-conjugated horseradish peroxidase with diaminiobenzidine as substrate (Vectastain Elite ABC Kit, Burlingame, CA, USA).

Samples demonstrating tumor cell localization of the antibody stains were classified as positive for protein expression. A hepatobiliary pathologist (OC) inspected each set of specimen cores and also rated the intensity of immunohistochemical staining on a 3-point scale with 1 = mild, 2 = moderate, and 3 = high. The cellular location of antibody staining (cytoplasm, membrane, or nucleus) was also recorded.

### Statistical Methods

Associations between protein expression and clinical data were analyzed by the Chi-square test or Fisher's exact test if appropriate. The time to event was defined as the number of months from the incidence date to the date of last follow-up or death due to any cause. Survival curves were estimated by the Kaplan-Meier (K-M) method and compared between groups on the basis of the log-rank test. Adjusted and unadusted hazard ratios (HR) and 95% confidence intervals (CI) were calculated using Cox proportional hazards regression modeling. A p-value of <0.05 was considered statistically significant. All statistical tests were performed using JMP Pro 9.0.2 64-bit Edition (SAS Institute Inc., Cary, NC).

## Results

### Patient characteristics

Immunohistochemical analysis was successfully completed on all 157 tumor specimens. Patient demographic and clinicopathologic characteristics are summarized in Table 1.

**Table 1:** Summary of SEER Reported Characteristics for 169 patients with HCC.

Characteristic	Number
Age Groups (years)	
<30	0
30–34	3
35–39	2
40–44	6
45–49	16
50–54	30
55–59	26
60–64	21
65–69	21
70–74	17
75–79	9
80–84	4
85–89	4
Gender (female/male)	46/113
Tumor Size	
< = 5 cm	80
>5 cm	71
Unknown	8
Tumor Grade	
1	37
2	58
3	28
4	5
Unknown	31
Stage (I/II/III/IV)	
I	110
II	32
III	1
IV	14
Unstaged	2
Alphafetoprotein Level	
>20 ng/ml	46
< = 20 ng/ml	28
Undetermined	21

### Immunohistochemical Results

**Figure 1:**
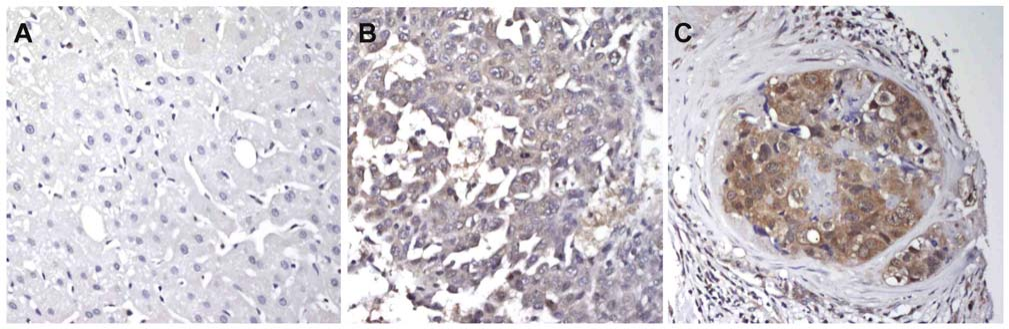
Photomicrographs of HCC tissue specimens stained using anti-CKA antibody. Images magnified at 200×. A) Normal liver tissue demonstrates an absence of immunohistochemical staining. B) Corresponding HCC tumor specimen from the same patient demonstrates mild cytoplasmic staining of moderately-differentiated tumor cells. C) Moderately-differentiated HCC nested within an area of fibrosis. The tumor cells demonstrate moderate cytoplasmic and nuclear staining.

Antibody staining for CKA was evident in 55 (35%) tumor specimens with moderate to high staining intensity in 15 (10%) of the samples. Immunohistochemical staining was localized to the cytoplasm in 36/55 of the CKA-positive tumor specimens, and to a combination of cytoplasm and nucleus in the remainder. Representative tissue staining patterns for anti-CKA antibody are shown in Figure 1. Tests of association between CKA staining and clinicopathological variables are summarized in Table 2. Notable was a borderline significant difference in tumor CKA expression across cancer stage.

**Table 2:** Distribution of CKA Positive Tumors in Groups Defined by Clinicopathological Variables.

Variable	Grouping	CKA		p value
		Positive	Negative	
Age (years)	<65	38	66	0.86
	> = 65	19	36	
Tumor Size	< = 5 cm	24	56	0.30
	>5 cm	30	41	
	Unknown	3	5	
Tumor Grade	Well-differentiated	9	28	0.09
	Moderately-differentiated	28	30	
	Poorly-differentiated	11	17	
	Undifferentiated-anaplastic	1	4	
	Unknown	8	23	
Stage	I	33	77	0.08
	II	17	15	
	III	0	1	
	IV	6	8	
	Unstaged	1	1	
AFP (ng/ml)	>20 ng/ml	17	29	0.25
	< = 20 ng/ml	5	23	
	Not documented	6	15	

Antibody staining for HK2 was evident in 71 (45%) tumor specimens with moderate to high staining intensity in 23 (15%) of the samples. Immunohistochemical staining localized to the cytoplasm in 63/71 of HK2-positive tumor specimens, and to a combination of cytoplasm and cell membrane in the remainder. Tests of association between HK2 staining and clinicopathological variables are summarized in Table 3. Significant differences in tumor HK2 expression were noted across cancer stage and tumor grade.

**Table 3:** Distribution of HK2 Positive Tumors in Groups Defined by Clinicopathological Variables.

Variable	Grouping	HK2		p value
		Positive	Negative	
Age (years)	<65	45	59	0.32
	> = 65	29	26	
Tumor Size	< = 5 cm	32	48	0.10
	>5 cm	36	35	
	Unknown	6	2	
Tumor Grade	Well-differentiated	8	29	0.007
	Moderately-differentiated	29	29	
	Poorly-differentiated	15	13	
	Undifferentiated-anaplastic	3	2	
	Unknown	19	12	
Stage	I	41	69	0.001
	II	23	9	
	III	0	1	
	IV	8	6	
	Unstaged	2	0	
AFP (ng/ml)	>20 ng/ml	23	23	0.20
	< = 20 ng/ml	10	18	
	Not documented	12	9	

Tumor immunohistochemical staining for HK2 was significantly associated with tumor staining for CKA (odds ratio 6.1, 95% CI 2.95–12.65, p<0.0001) (Table 4).

**Table 4:** Contingency Table of HK2 and CKA Expression.

	HK2 Positive	HK2 Negative	Row Totals
**CKA Positive**	41	16	57
**CKA Negative**	33	69	102
**Column Totals**	74	85	159

### Survival Analysis

The mean duration of longitudinal follow-up in 157 patients was 48 months (range 0 to 294 months). Stage at the time of cancer diagnosis was significantly associated with different patterns of overall survival (Log-rank p<0.0001) with median survival times of 65, 12, 5, and 6 months, for stage I, II, III, and IV respectively. Tumor size less than or equal to 5 cm was also significantly associated with increased overall survival (Long-rank p<0.001) with median survival times of 86 months and 26 months in patients with tumors < = 5 cm and >5 cm, respectively. Patient age and tumor grade were not significantly associated with different survival patterns.

### Survival Associated with Tumor CKA and HK2 Expression

**Figure 2:**
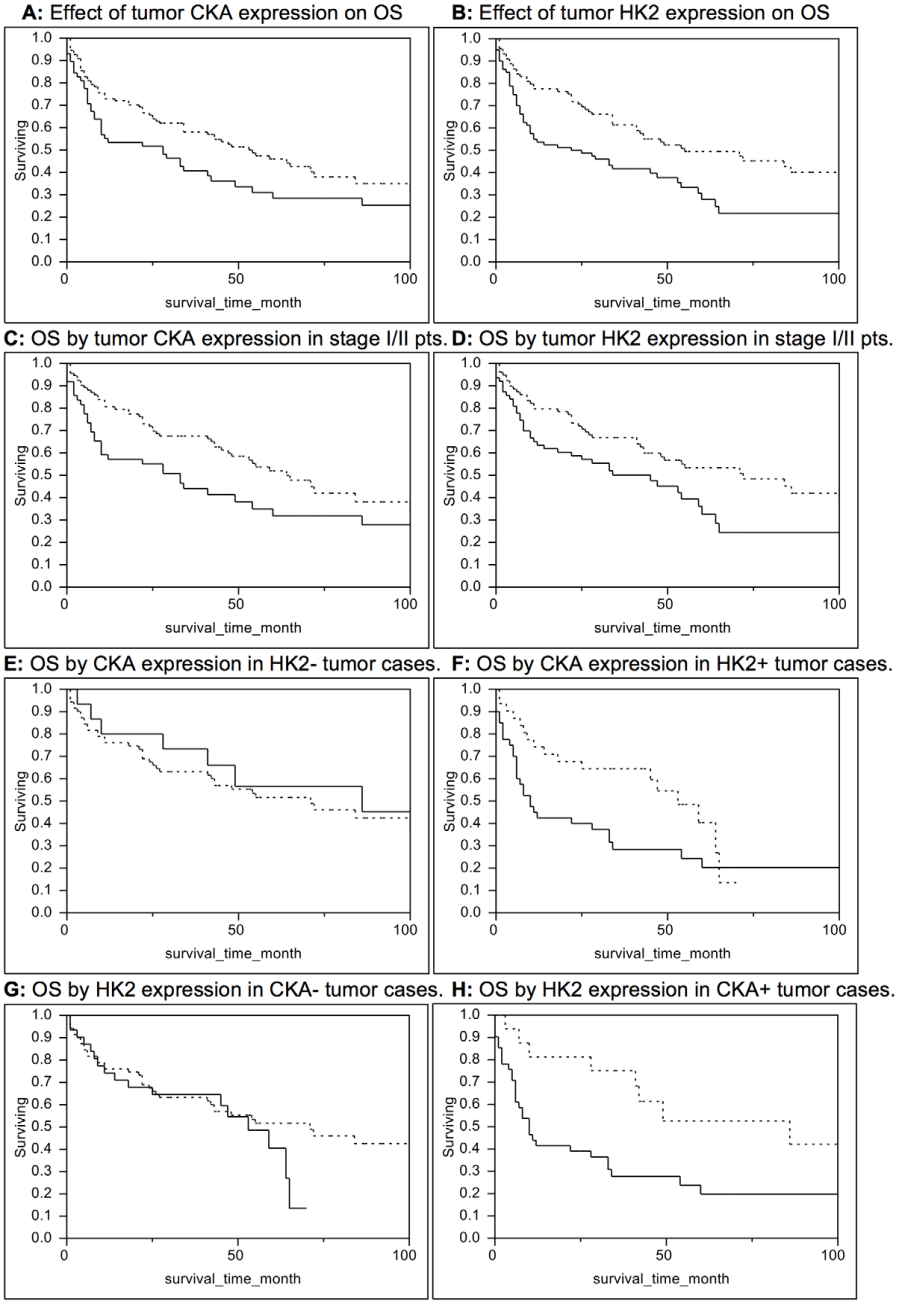
Patterns of overall survival (OS) based on tumor immunohistochemical expression of CKA and HK2. Solid lines represent immunohistochemistry-positive cases while dotted lines represent immunohistochemistry-negative cases. A) OS was significantly worse for patients with CKA-positive tumors relative to patients with CKA-negative tumors (Log-Rank test p = 0.03). B) OS was also significantly worse for patients with HK2-positive tumors relative to patients with HK2-negative tumors (Log-Rank test p = 0.003). C) Among stage I and II HCC patients, OS was significantly worse for patients with CKA-positive tumors relative to patients with CKA-negative tumors (Log-rank p = 0.03). D) Among stage I and II HCC patients, OS was also significantly worse for patients with HK2-positive tumors relative to patients with HK2-negative tumors (Log-rank p = 0.02). E) Among patients with HK2-negative tumors, OS did not differ significantly on the basis of CKA expression (Log-Rank test p = 0.53). F) Among patients with HK2-positive tumors, OS was significantly worse in patients whose tumors were also CKA-positive (Log-Rank test p = 0.04). G) Among patients with CKA-negative tumors, OS did not differ significantly on the basis of HK2 expression (Log-Rank test p = 0.35). H) Among patients with CKA-positive tumors, OS was significantly worse in patients whose tumors were also HK2-positive (Log-Rank test p = 0.01).

Tumor CKA expression was significantly associated with increased mortality (Log-rank p = 0.03), with a median survival time of 28 months for 55 patients with CKA-positive tumors compared to 59 months for 102 patients with CKA-negative tumors (Figure 2A). The unadjusted HR corresponding to the detection of tumor CKA expression was 1.59 (95%CI 1.04–2.41). A greater effect on survival was associated with moderate to high intensity tumor staining for CKA (HR 4.28, 95% CI 2.29–7.44).

Tumor HK2 expression was also associated with increased mortality (p = 0.003), with a median survival time of 28 months for 71 patients with HK2-positive tumors compared to 72 months for 86 patients with HK2-negative tumors (Figure 2B). The unadjusted HR corresponding to the detection of tumor HK2 expression was 1.86 (95% CI 1.23–2.83). Moderate to high intensity tumor staining for HK2 was also positively associated with mortality (HR 2.19, 95% CI 1.24–3.63).

### Survival Patterns Among Early Stage (I and II) Patients

Tumor CKA expression was associated with increased mortality in an analysis limited to the 142 patients with stage I and II HCC (Log-rank p = 0.03). The median survival in 49 stage I and II patients with CKA-positive tumors was 33 months versus 64 months in 93 stage I and II patients with CKA-negative tumors (Figure 2C). On univariate analysis, the HR associated with tumor CKA expression in stage I and II HCC was 1.63 (95% CI 1.03–2.55).

Survival patterns among stage I and II HCC patients also differed significantly on the basis of tumor HK2 expression with a median survival time of 45 months in 63 stage I and II patients with HK2-positive tumors versus 72 months in 79 stage I and II patients with HK2-negative tumors (Log-rank p = 0.02) (Figure 2D). The unadjusted HR associated with tumor HK2 expression in cases of stage I and II cancer was 1.70 (95% CI 1.09–2.66).

### Survival Patterns Associated with Tandem Expression of CKA and HK2

Among the 86 patients with HK2-negative tumors, overall survival did not differ significantly on the basis of whether tumors were also CKA-positive or CKA-negative (respective median survival times 86 versus 71 months, p = 0.53) (Figure 2E). However, among 71 patients with HK2-positive tumors, increased mortality was observed in patients whose tumors were also CKA-positive (Figure 2F). In this group, median duration of survival in 40 patients with CKA-positive tumors was 10 months versus 53 months in 31 patients with CKA-negative tumors (p = 0.04). The HR associated with tumor CKA expression in patients with HK2-positive tumors was 1.84 (95% CI 1.02–3.43).

Among the 102 patients with CKA-negative tumors, survival patterns did not differ significantly on the basis of whether tumors were also HK2-positive or HK2-negative (respective median survival times: 53 versus 71 months, p = 0.35) (Figure 2G). However, among 55 patients with CKA-positive tumors, significantly increased overall mortality was observed among patients whose tumors were also HK2-positive (Figure 2H). The median duration of survival in 40 patients with CKA-positive/HK2-positive tumors was 10 months as compared to 86 months in 15 patients with CKA-positive/HK2-negative tumors (p = 0.01). The HR associated with tumor HK2-expression among patients with CKA-positive tumors was 2.82 (95% CI 1.30–7.03).

### Multivariable Analysis

Cancer stage (p = 0.003), tumor size < = 5 cm (p = 0.024), and tumor HK2 expression (p = 0.047) exhibited significant independent effects on overall survival in a proportional hazards regression model that also adjusted for patient age, gender, and tumor grade. In this model, the HR associated with tumor HK2 expression was 1.62 (95% CI 1.00–2.60). In a model that adjusted for patient age, gender, tumor grade, and tumor CKA expression, only cancer stage (p = 0.002) and tumor size (p = 0.033) demonstrated significant effects on overall survival.

## Discussion

This study reports an interesting heterogeneity among patients with regards to tumor immunohistochemical expression of HK2 and CKA. Tumor expression of HK2 differed significantly across tumor grade and cancer stage and was associated with poorer overall survival. Tumor expression of CKA, while not as strongly associated with other clinicopathologic variables, was also associated with less favorable patterns of survival. The survival effects associated with these immunohistochemical markers remained significant in analyses restricted to patients with early stage (I and II) HCC. Based on these findings, we discuss several plausible mechanisms by which these specific enzymes may be contributing to a more aggressive cancer phenotype.

Hyperglycolysis, along with increased HK2 expression and activity, has been reported in a variety of cancers [4]. PET imaging studies involving viral-induced woodchuck hepatomas suggest glycolytic activity to vary among liver tumors in association with the levels of HK2 activity [12]. In other cancers, expression of HK2 has been strongly associated with increased tumor biologic aggressiveness [27]–[29]. An association between HK2 expression, cancer stage, and survival found in the current study might therefore suggest that abnormal glycolysis is a feature of biologically aggressive tumors in HCC.

The tendency for malignant tumors to exhibit increased glycolytic activity under conditions suitable for oxidative phosphorylation, a phenomenon known as the Warburg effect, has been hypothesized to confer tumor cells with a survival advantage [2]. Specifically, glycolysis produces lactate, which may not only increase tumor antioxidative capacity but also reduce extracellular pH that in turn can expedite extracellular matrix remodeling, dampen host defenses, and facilitate tumor invasion and metastasis. In conditions of low oxygen tension, hypoxia-indicuble factor-1 alpha (HIF-1) has also been shown to upregulate HK2 expression and stimulate the proliferation of hepatoma cells [30]. Co-expression of HIF-1 and HK2 has also been found to disproportionately localize in the central portions of hepatomas as well as to areas surrounding tumoral necrosis [31], [32]. Altogether, these results implicate glycolysis in the adaptation of liver tumors to a hostile stromal environment. The role of glycolysis in sustaining tumor progression may therefore underly the association between tumor HK2 expression and patient mortality observed in the current study.

Currently, FDG PET is the only method available for non-invasively measuring tissue glycolysis in-vivo. Tumor FDG uptake on PET has been correlated with risk of tumor recurrence in patients undergoing hepatic resection for HCC [14], [33], [34]. Tumor FDG uptake has also been shown to predict recurrence-free survival in patients undergoing liver transplantation for HCC [35]. Furthermore, HCC tumor differentiation has been shown to correlate with tumor FDG uptake [34]. The association of tumor HK2 expression with HCC grade, stage, and overall survival in the current study helps to explain the results of these clinical FDG PET studies, given that the cellular retention of FDG is mediated by HK2.

Tumor CK expression was also significantly associated with less favorable survival outcomes in this study. CK catalyzes the production of phosphocholine, a substrate used in the Kennedy (citidine diphosphocholine-choline) pathway to synthesize phosphatidylcholine and other membrane phospholipids [36]. Increased CK expression, along with increased intracellular levels of phosphocholine, occurs in a variety of cancers and is ostensibly related to the rate of cellular proliferation in tumors [8], [37]–[41]. Thus, the association between tumor CK expression and survival in this study may be explained on the basis of tumor growth. However, several recent studies have not substantiated a direct relationship between tumor proliferative activity and choline metabolism [10], [42]–[44]. An alternative explanation for why increased CK activity can lead to more aggressive tumors is the potential role of phosphocholine as a second-messenger in mitogenic signaling. Specifically, the production of phosphocholine appears necessary for the activation of Raf-1 in the mitogen activated protein kinase (MAPK) pathway [45], as well as the activation of protein kinase B (PKB/Akt) in the phosphatidylinositol 3-kinase (PI3K)-Akt pathway [46]. This role of phosphocholine in mitogenic signal transduction may therefore also serve as another potential explanation for the association between tumor CKA expression and increased mortality found in the current study.

Tumor HK2 expression was a weaker predictor of survival than tumor size and cancer stage in multivariable analysis. The impact of tumor CKA expression was also mitigated after adjustments for other clinicopathologic variables. Therefore, these immunohistochemical markers may have limited clinical value for patient risk stratification beyond the clinical parameters already used to assess patient prognosis. Nonetheless, the current study provides evidence that glycolysis and choline metabolism are biologically relevant to clinical outcomes in HCC, which should encourage further investigation of these metabolic pathways as potential therapeutic targets in HCC.

In this study, tumor HK2 expression status identified a subset of patients with less favorable prognosis among patients with CKA-positive tumors. In turn, tumor CKA expression identified patients with worse prognosis among patients with HK2-positive tumors. The biologic basis of these observations may be uncertain since the relationship between glucose metabolism and choline metabolism in cancer is not yet well understood. In breast cancer cells, CKA down-regulation has been shown to exert little effect on cellular FDG metabolism, suggesting that glucose metabolism may not be coupled to choline metabolism [47]. This raises the possibility that glucose metabolism and choline metabolism are independently advantageous to tumor cells. A recent PET imaging trial comparing FDG PET with fluorine-18 labeled choline (FC) PET for the detection of HCC reported that well-differentiated tumors are more likely to be detected by only FC PET, while less differentiated tumors may be detected by either PET technique with similar efficacy [13]. In view of the current study results, it may be hypothesized that livers tumors showing abnormalities on both FDG and FC PET are potentially more aggressive and lethal.

One limitation of this study is that the use of microscopy arrays limits the assessment of HK2 and CKA expression to very small portions of tumor. It was not possible to confirm the clinical tumor grade of these samples given the amount of tissue that was analyzed. Differences in the immunohistochemical expression of these markers may also exist across different tumor regions. Such intratumoral heterogeneity in immunohistochemical expression was evident in a study of xenograft hepatomas, where HK2 expression was noted to differ between the tumor center and periphery [32]. Because of the limited sampling involved, the current study may underestimate the overall magnitude of CKA and HK2 expression in HCC tumors.

Another potential limitation to this study is that the data available from cancer registries is not comprehensive from a clinical standpoint. A number of clinically important variables, such as serologic markers of liver disease severity and viral hepatitis status, could not be examined since this data was not routinely abstracted by the cancer registries. Moreover, information on tumor recurrence and subsequent treatment was not available for analysis.

### Conclusions

Immunohistochemical expression of HK2 and CKA in tumors was associated with poor survival in HCC. These prognostic effects could be seen even in analyses limited to early stage (I and II) cases. Such associations support speculation that glycolysis and choline metabolism are involved in the biologic progression of HCC into more aggressive and lethal cancer phenotypes.
